# Method to isolate polyribosomal mRNA from scarce samples such as mammalian oocytes and early embryos

**DOI:** 10.1186/1471-213X-11-8

**Published:** 2011-02-15

**Authors:** Sara Scantland, Jean-Philippe Grenon, Marie-Hélène Desrochers, Marc-André Sirard, Edward W Khandjian, Claude Robert

**Affiliations:** 1 Laboratoire de génomique fonctionnelle du développement embryonnaire, Centre de recherche en biologie de la reproduction, Pavillon Comtois, Faculté des sciences de l'agriculture et de l'alimentation, Université Laval, Québec, G1V 0A6, Canada; 2 Laboratoire de génomique et protéomique animales, Centre de recherche en biologie de la reproduction, Pavillon INAF, Département des sciences animales, Faculté des sciences de l'agriculture et de l'alimentation, Université Laval, Québec, G1V 0A6, Canada; 3 Neurobiologie cellulaire, Centre de recherche Robert-Giffard, Département de psychiatrie et de neurosciences, Faculté de Médecine, Université Laval, Québec, G1V 0A6, Canada

## Abstract

**Background:**

Although the transcriptome of minute quantities of cells can be profiled using nucleic acid amplification techniques, it remains difficult to distinguish between active and stored messenger RNA. Transcript storage occurs at specific stages of gametogenesis and is particularly important in oogenesis as stored maternal mRNA is used to sustain *de novo *protein synthesis during the early developmental stages until the embryonic genome gets activated. In many cases, stored mRNA can be several times more abundant than mRNA ready for translation. In order to identify active mRNA in bovine oocytes, we sought to develop a method of isolating very small amounts of polyribosome mRNA.

**Results:**

The proposed method is based on mixing the extracted oocyte cytoplasm with a preparation of polyribosomes obtained from a non-homologous source (*Drosophila*) and using sucrose density gradient ultracentrifugation to separate the polyribosomes. It involves cross-linking the non-homologous polyribosomes and neutralizing the cross-linking agent. Using this method, we show that certain stages of oocyte maturation coincide with changes in the abundance of polyribosomal mRNA but not total RNA or poly(A). We also show that the abundance of selected sequences matched changes in the corresponding protein levels.

**Conclusions:**

We report here the successful use of a method to profile mRNA present in the polyribosomal fraction obtained from as little as 75 mammalian oocytes. Polyribosomal mRNA fractionation thus provides a new tool for studying gametogenesis and early development with better representation of the underlying physiological status.

## Background

Gametogenesis and embryonic development in mammals involve several major cellular events marked by an unusual mode of messenger RNA management. In nearly all animal species, mRNA molecules are stored in the developing oocyte until use during maturation or after fertilization [1-8]. These stored mRNAs direct protein synthesis during the period of transcriptional silence, which begins when the germinal stage oocyte reaches its full size [9-13] and lasts until embryonic genome activation [14-16]. In cattle, this size is approximately 120 μm within a follicular antrum 3-5 mm in diameter [10,15,17]. During the period covering the remaining follicular development (i.e. from 3 to 25 mm in antral diameter), the post-LH-surge oocyte maturation, fertilization and the onset of embryonic genome activation, very little genomic transcription occurs. It is generally believed that transcript storage begins in the early stages of oogenesis and may thus last for several weeks. It is also believed that the transcripts are stored in a particulate form [18] and lack the poly(A) portion, although the latter detail remains the subject of debate. It has been reported that shortening the poly(A) tail to less than 50 nucleotides stabilizes the mRNA molecule and keeps it from being either degraded or translated [19].

So far, little is known about the molecular mechanisms underlying the steps that occur during this transcriptional silencing period. Early development is characterized by major fluctuations in the abundance of total and messenger RNA [2,20], with specific waves of maternal RNA degradation [14,21]. These observations have led to the belief that measurement of messenger abundance provides little useful information about cells that are storing RNA, since it does not distinguish between mRNA that is 1) stabilized and stored and thus not contributing to any cellular function; 2) recruited and on its way to degradation, not contributing to the translation process and 3) recruited and being translated in *de novo *protein synthesis. In order to avoid the contribution of the stored or decaying molecules to the mRNA abundance measurements, we seek to provide a mean to isolate the mRNA population bound to the translation apparatus. Messenger RNAs engaged in translation are found to be bound by ribosomes throughout the cytosol either freely or attached to the cytoskeleton while dormant or stored transcripts are accumulated in diverse forms of ribonucleoprotein complexes and particles [22]. It is also well known that actively translated messengers are bound by multiple ribosomal units [23-26].

The composition of these different particles makes it possible to fractionate them by density gradient. Profiling of polyribosomal mRNA through standard sucrose gradient fractionation procedure requires considerable starting material (e.g. 200 μg of total RNA [27]). A recent publication reports the development of a method suitable for input material not fewer than five *Xenopus *laevis oocyte, eggs or early embryos [28]. Considering the *Xenopus *oocyte contains about 15,000 times more total RNA comparatively to the bovine counterpart (respectively 6 μg [29] and 340 pg [20]), the method still requires too much input for work on mammalian early development. The relative scarcity of mammalian oocytes, egg and embryonic tissues is another impediment to increasing understanding of mammalian gametogenesis and early development, since the choice of methods suitable for handling such minute quantities of material is severely limited. Pre-amplifying the entire transcriptome offers the possibility of studying the fluctuations in transcript abundance in these tissues. Minute amounts of initial RNA can be amplified with success [30-32] and provide sufficient output for high throughput approaches such as microarrays [33,34] or systematic deep sequencing (RNAseq) [35,36].

To our knowledge, the isolation of polyribosomes from mammalian oocytes or early embryos has been reported only twice and resulted in limited success [37,38]. The methodology used by De Leon and colleagues [29] involves spiking a small sample with a large amount of genetically homologous material to confirm the polyribosomal nature of the RNA molecules being studied. However, the inability to distinguish between the spike and the sample prevented identification of oocyte/embryo mRNA molecules. In contrast, the approach used by Potireddy and colleagues [28] allowed mRNA identification but could not confirm the polyribosomal nature of the isolated fraction nor exclude the presence of non-polyribosomal contaminants. We therefore sought to combine the advantages of each method by devising means of confirming the polyribosomal nature of the extracted mRNAs while maintaining the possibility of identifying them and determining their relative abundance.

## Results

### Preparation of the inert carrier

In order to develop a polyribosomal isolation method that could be performed with very small quantities of sample material, we used an RNA carrier fraction. Spiking the bovine sample with polyribosomes from a non-homologous organism (i.e. *Drosophila*) is helpful as long as there is a way to prevent interference with downstream transcript identification.

**Figure 1:**
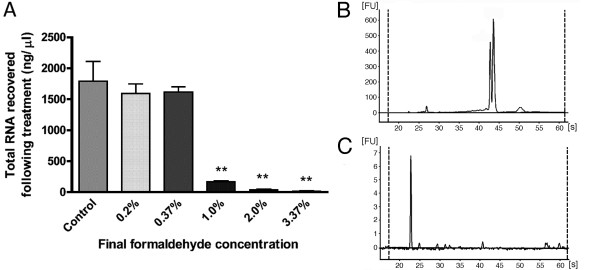
**Effect of cross-linking on recovery of RNA extracted from *Drosophila *SL2 cells**. A) Total RNA recoverable from cell extracts (the clarified supernatant) after cross-linking with various concentrations of formaldehyde; Micro-electrophoretic profile of the total RNA recovered from B) untreated cell extract; C) cell extract treated with 3.37% formaldehyde.

Formaldehyde was used to cross-link RNA and proteins from the drosophila SL2 cell extracts in order to produce a range of materials that might function as carriers. To determine the optimal concentration of formaldehyde that would provide a useful carrier and minimize downstream interference, a dose-response experiment was done. At lower concentrations (i.e. 0.2% and 0.37%), cross-linking was slight, as indicated by the recovery of almost all of the initial RNA in sucrose density gradient fractions. At a concentration of 1% formaldehyde, approximately 10% of the RNA could be recovered while at the highest concentration tested (3.37%), less than 1% of the initial RNA input could be recovered (Figure 1A). The micro-electrophoretic profile confirmed the extremely low level of RNA recovered following the 3.37% formaldehyde treatment (Figure 1B-C). This latter treatment was used and an additional step was included to neutralize excess formaldehyde prior to adding the SL2 cell carrier polysome preparation to the experimental samples.

**Figure 2:**
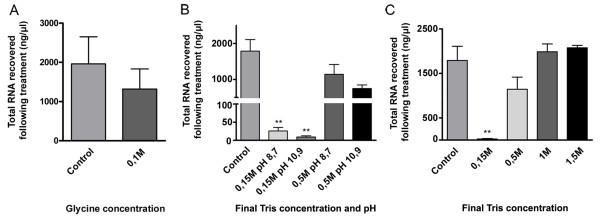
**Neutralization of formaldehyde in *Drosophila *SL2 cell extract**. Total RNA recovered from cell extracts (the clarified supernatant) treated with 3.37% formaldehyde, quenching the reaction using A) Glycine at pH 8.7; B) Tris at different concentrations and pH; C) Tris at a broad range of concentrations at pH 8.7

**Figure 3:**
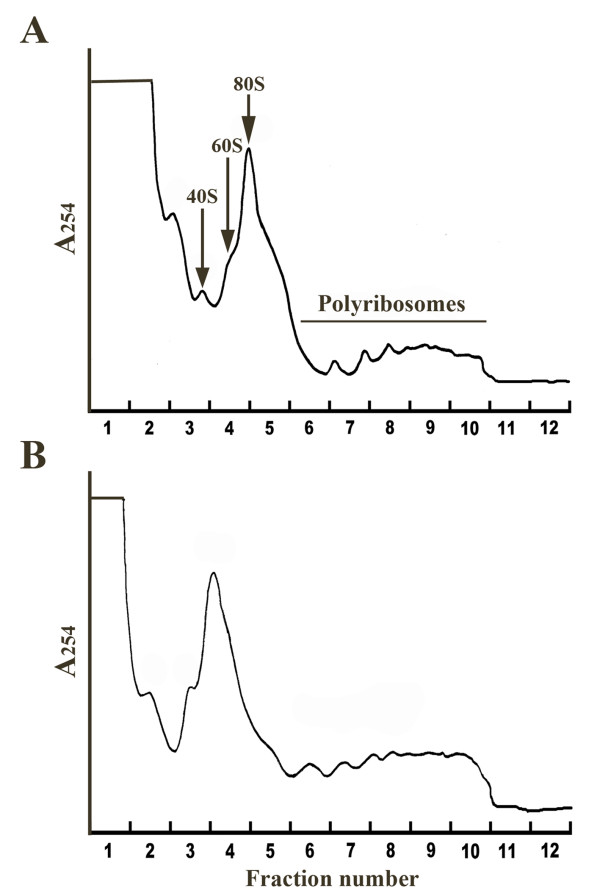
**Distribution of *Drosophila *SL2 polyribosomes in sucrose density gradient fractions as detected by UV absorbance**. A) Untreated cytoplasmic extract; B) Cytoplasmic extract treated with 3.37% formaldehyde and quenched with 1 M Tris. Ribosomal subunits (40S and 60S), monoribosomes (80S), polyribosomes and corresponding fraction numbers are indicated. Top of gradient corresponds to fraction 1.

Glycine, used routinely to titrate free formaldehyde and used in this study at the commonly used concentration of 0.1 M [39] was not entirely effective at neutralizing this cross-linking agent (Figure 2A). Since tris-hydroxymethylaminomethane molecule (THAM or Tris) has been suggested for this purpose [40], tests were conducted to determine the conditions under which it would efficiently inactivate residual formaldehyde in SL2 cell extract. The impact of pH was also tested since polyribosome extraction was done at a lower pH than in the Sutherland study. The impact of pH was found not significant (Figure 2B). At a concentration of 0.5 M, Tris was found comparable to 0.1 M glycine and thus could not be considered more efficient. At concentrations of 1 M and 1.5 M, Tris did neutralize the residual formaldehyde completely (Figures 2B and 2C). Figure 3 shows the effect of the RNA carrier preparation protocol on the distribution of polyribosomes in the sucrose density gradient fractions of the *Drosophila *SL2 cell extract. The crosslinking treatment did not interfere with polyribosome profiling as both treated and control samples show very similar profiles (Figure 3).

### Validation of the polyribosomal nature of the isolated RNA

**Figure 4:**
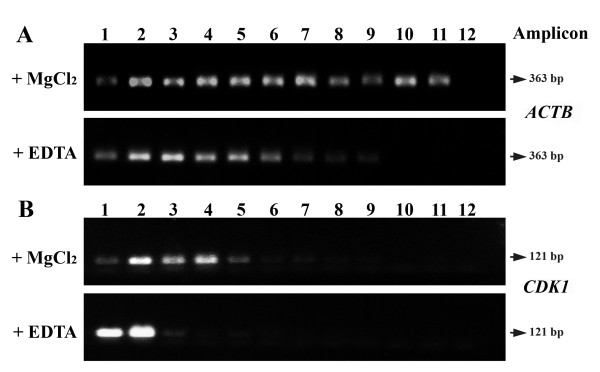
**Confirmation that the high-sedimenting materials contain polyribosomes**. Cells were lysed and fractionated in the presence or absence of EDTA. RT-PCR was used to amplify the *ACTB *and *CDK1 *sequences from RNA isolated respectively (A) from granulosa cells and (B) from GV-stage oocytes. Top of gradient corresponds to fraction 1.

Since polyribosomes are stabilized by Mg^2+ ^ions, addition of EDTA causes their dissociation into ribosomal subunits and the release of messenger RNA. Cytoplasmic extracts were therefore fractionated in the presence or absence of EDTA. In order to observe this in the absence of the *Drosophila *polyribosome preparation, a suitable quantity of granulosa cells was processed. Standard RT-PCR of genes *ACTB *(for granulose cells) and *CDK1 *(for oocytes) was used as means of comparing RNA abundance in the collected fractions. For the two cell types, the presence of EDTA caused a shift in RNA abundance towards the fractions containing low sedimentation coefficient materials, thus confirming the polyribosomal nature of the higher sedimenting fractions (Figure 4).

### Interference of *Drosophila *polyribosome RNA with microarray hybridization signals

Interference by the exogenous carrier RNA with the density-gradient fractionation of the oocyte RNA was minimal. We decided to determine if this was true for microarray hybridization signals. Samples prepared from purified bovine oocyte RNA and from purified *Drosophila *SL2 RNA were labelled with different fluorophores and hybridized on the same microarray. Table 1 summarizes the proportion of microarray features that generated positive fluorescent signals above the background threshold. In spite of its phylogenetic distance from cattle, *Drosophila *RNA generated one third of positive spots that bovine RNA did at 55°C. Increasing the hybridization temperature by 5°C resulted in a significant loss in *Drosophila *positive signals and at 65°C, no *Drosophila *signal above background was detected. However, at 65°C, almost half of the bovine signals were also lost. The intermediate temperature seemed to be an acceptable compromise since only 6% of the spots generated positive, but very weak signals from *Drosophila *samples, while the fluorescence values from the bovine samples were still clearly above background. By comparison to the least stringent condition (55°C), 77% (47/61) of the bovine signals were kept when microarrays were hybridized at 60°C (Table 1).

**Table 1:** Proportion of detected microarray signals above threshold

	Hybridization Temperature (°C)		
	
Species	55°C	60°C	65°C
*Bos taurus*	61%	47%	36%
*Drosophila melanogaster*	20%	6%	0%

### Reproducibility of the polyribosome RNA extraction method

**Figure 5:**
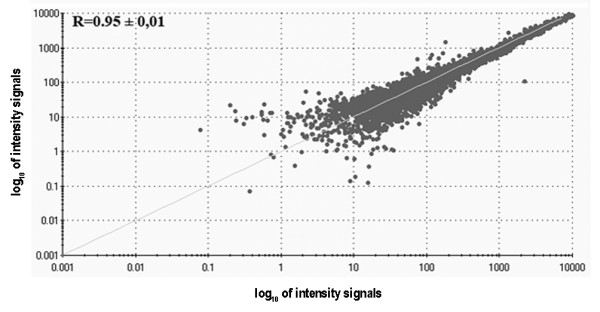
**Reproducibility of the polyribosome mRNA extraction method**. Distribution of microarray signal intensities obtained from two independent biological replicates. The mean correlation value of all three replicates is indicated.

We tested the reproducibility of the entire method by comparing the results obtained from biological replicates. Three oocyte pools were thus fractionated and analyzed separately using the density gradient method and microarray hybridization. The mean correlation value was found to be 0.95 ± 0.01, which clearly indicates that the procedure is robust (Figure 5).

### Validation of the method under different physiological conditions

**Figure 6:**
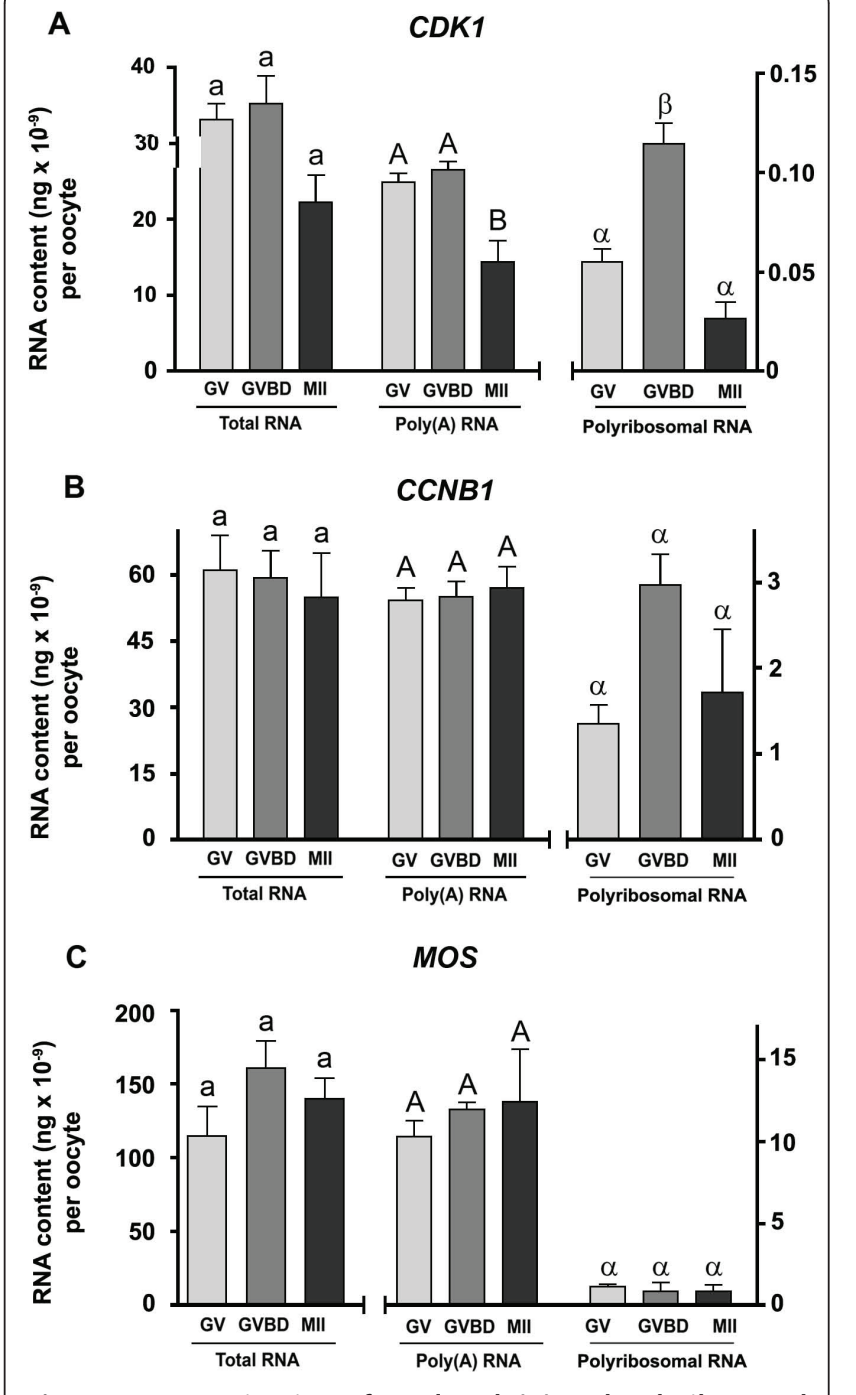
**Determination of total, poly(A) and polyribosomal RNA in oocytes at different stages of physiological maturation**. Quantitative RT-PCR was used to amplify the following sequences: A) *CDK1*; B) *CCNB1*; and C) *MOS*. GV = germinal vesicle; GVBD = germinal vesicle breakdown; MII = metaphase II. For each RNA population different superscript indicate statistically significant difference (p < 0.05).

The polyribosome fractionation protocol was tested with oocytes at different stages of maturation using microarray hybridization to measure the abundance of RNA sequences representing known key factors in the control of oocyte maturation. Quantitative RT-PCR was used since probes for some of the chosen factors were absent on our microarray. The selected sequences correspond to components of maturation promoting factor, namely cyclin B1 (*CCNB1*), cyclin-dependant kinase 1 (*CDK1*) and Moloney sarcoma oncogne (*MOS*), which is part of the cytostatic factor required at the MII stage. Total RNA (targeted using random primers during the RT reaction), poly(A) (targeted using an oligo-dT during RT) and polyribosomal sub-fractions were measured. The abundance of these RNA types varied significantly between the different tissue types (Figure 6), indicating clearly that the stage of maturation of the oocyte sample has an impact on the distribution of the RNA.

### Potential implications for protein levels

**Figure 7:**
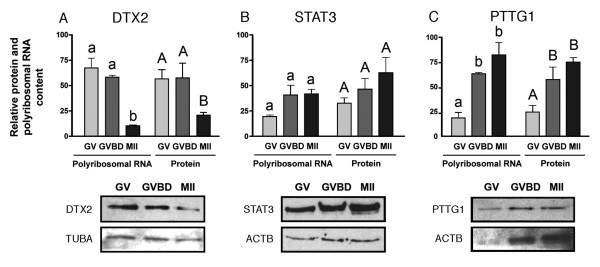
**Relative abundance of selected polyribosomal mRNA sequences and corresponding proteins**. Immunoblots were prepared from oocytes at the GV, GVBD and MII stages. The three candidates were selected based on microarray data. A) Deltex 2 (DTX2); B) Signal transducer and activator of transcription 3 (STAT3); C) Pituitary tumour-transforming gene 1 (PTTG1). β-actin (ACTB) and α-tubulin (TUBA) were used as indicators of protein loading. For each component different superscript indicate statistically significant difference (p < 0.05).

Since mRNA associated with polyribosomes is presumed actively translated, levels of protein corresponding to the selected factors were measured. Microarray gene entries corresponding to polypeptide sequences for which commercial antibodies were available were selected. Standard housekeeping candidates ACTB and TUBA were used as internal standards. Due to the requirement of oocyte maturation for extensive reorganization of the cytoskeleton, these standard housekeeping candidates were found to be unstable and were therefore considered solely as a control of sample loading and not used for data normalization. The fluctuations in polyribosomal mRNA levels observed between maturation stages closely matched protein levels for all candidates, as shown in Figure 7.

## Discussion

The need to develop a procedure for isolating and studying polyribosomal mRNAs from mammalian gametes and early embryos arose from the peculiarity of these cells. The collection of mammalian oocytes and early stage embryos is challenging and resource-intensive. Therefore, samples rarely contain more than 100 oocytes/embryos. Their scarcity imposes a method suited to handling minute quantities of material. For instance, protein profiling and identification of differentially expressed candidates requires several hundreds to thousands of mammalian oocytes or early embryos [41-44]. Similarly, these tissues do not provide a much better source of RNA considering for example that a single Xenopus oocyte contains about 6 μg of total RNA [29] comparatively to only 340 pg in the bovine [20]. Nonetheless, the wide array of amplification procedures now available has made it possible to focus on the transcriptome rather than the proteome. Study of the transcriptome generally assumes that mRNA levels are indicative of cellular status and reflect corresponding protein levels. However, this assumption does not apply to cells containing large amounts of stored mRNA, such as mammalian oocytes and early blastomeres. In these cases, mRNA bound to polyribosomes and therefore likely being translated is considered a better indicator of gene activity and developmental stage.

The first important step for polyribosomal RNA extraction is thorough lysis of the cells. The greatest difficulty encountered when working with oocytes or pre-hatching embryos is the challenge of breaking down the zona pellucida. This porous glycoprotein coat is composed of a dense net of fibril bundles that evolves during oocyte maturation and fertilization [45]. The bovine zona pellucida is particularly resistant, with a thickness averaging 26.9 μm compared to 14.5 μm for ovine and 11.4 μm for murine oocytes [46]. Since the lysis buffer used in the polyribosome isolation procedure is relatively mild to disrupt the cytoplasmic membrane and liberate intact polyribosomes, an additional treatment was required to efficiently disrupt the sturdy zona pellucida to liberate the cellular contents. We found that freeze/thaw cycles, effective for mouse oocytes [38], are ineffective against the bovine zona pellucida and that digestion with pronase produced irreproducible results due to residual protease activity. The previously used acidic (pH 2.1 to 2.5) Tyrode buffer [37] was also tested, but changes in the granular appearance of the oocyte cytoplasm suggested disruption of the cytoskeleton, to which polyribosomes are believed to be bound [47]. Moreover, removal of the zona pellucida by acidic treatment has been reported to lead to embryo death and increased frequency of abnormalities in surviving embryos [48], suggesting damage to the embryo development program in which polyribosomes are involved. The only acceptable option appeared to be mechanical breakage of the zona with zirconia-silica beads in the presence of passive lysis buffer (Additional File 1). This approach seemed to work since the zona and its contents were completely dissolved within a few minutes.

To our knowledge, extraction of polyribosomes from oocytes and early embryos has been reported only twice [37,38]. In the first case, mouse liver polyribosomes acted as a carrier of polyribosomal mRNA extracted from [^3^H]-uridine-labelled mouse oocytes. This attractive method allows confirmation of the presence of oocyte polyribosomes, but does not allow any identification or even relative quantification of the associated mRNA, since it cannot be distinguished from that of the liver polyribosomes. The second study involved a method that allowed identification of the transcripts but could not confirm their polyribosomal nature nor exclude the presence of non-polyribosomal contaminants.

We have developed a method in which a heterologous carrier is used and which allows identification of extracted mRNA and confirmation of its polysomal nature. This involved determining optimal conditions for cross-linking the exogeneous polyribosomes. UV cross-linking was incomplete and prolonging the exposure led to RNA fragmentation (Additional File 2). Formaldehyde cross-linking, which binds more specifically via free amino groups [49] was found more effective than UV. Before adding the carrier preparation to the biological sample, it was necessary to neutralize the excess formaldehyde, which is normally done with glycine. A recent study of the efficiency of glycine suggests using stronger nucleophiles such as Tris or lowering the solution pH as quick and efficient means of quenching residual formaldehyde [40]. Tris was the preferred option, since it was not clear that lowering the pH would neutralize the formaldehyde without damaging the polyribosomes.

Using the method described here, the polyribosomal nature of the isolated bovine RNA can be inferred from its position in the sucrose gradient. Further validation was obtained using EDTA to disrupt the polyribosomes by sequestering Mg^2+^. The shift observed in the abundance of the amplified *CDK1 *fragment towards lower density fractions following this disruption is indicative of the polyribosomal nature of the bovine mRNA in the higher density fractions.

The strength of the approach used here was assessed by quantifying the abundance of selected mRNA of genes known to be involved in oocyte maturation. Following the luteinizing hormone surge, the oocyte resumes meiosis and undergoes a sequence of events involving germinal vesicle breakdown (GVBD), first polar body extrusion and a second arrest at the metaphase of the second meiosis (MII) in preparation for fertilization. It is known that maturation promoting factor (MPF), a heterodimer composed of cyclin B1 (CCNB1) and cyclin-dependant kinase 1 (CDK1, formerly known as p34^cdc2^), must be activated for meiosis to resume. Once meiosis reaches the MII stage, the role of the CSF is to halt cell cycle until the ovule is fertilized (For reviews, see [50,51]). We previously observed that in cattle, the GV-stage oocyte lacks the CCNB1 component but contains the CDK1 protein [52]. For activation of MPF, *CCNB1 *must be translated immediately after meiosis resumes but before germinal vesicle breakdown. Consistent with these observations, *CCNB1 *mRNA was found in the polyribosomal fraction at the GV stage, in addition to *CDK1 *mRNA. CSF is activated during a later stage of oocyte maturation prior to its involvement in arresting the cell cycle. However, it has been found that MOS is expressed readily during early stages of oocyte maturation, since it is involved in CCNB1 accumulation and displays an MPF stabilizing activity at the MII stage [53]. Following fertilization, both the MPF and CSF are rapidly degraded. Consistent with the activation of MPF, our results showed that levels of *CDK1 *mRNA and *CCNB1 *mRNA present on polyribosomes increased during the initial step of maturation while *MOS*, known to be involved throughout oocyte maturation, was equally present at all stages. This physiologically relevant profile was not observed when targeting total or poly(A)-bearing RNA. This is a strong confirmation that transcript abundance measured as total maternal mRNA pool need to be interpreted with care due to the contribution of the large contents of stored thus physiologically inert mRNAs.

Finally, we also investigated the proportionality between specific polyribosomal mRNAs and their corresponding protein products. By definition, polyribosomal RNA encodes protein to be newly synthesized. In contrast, the protein content of a candidate results from both its synthesis and turnover rates. Nevertheless, of the three mRNA sequences studied in parallel by Western blot, all showed a shift in total protein that matched the shift in their polyribosomal status, confirming the added value of polyribosome-bound mRNA studies in terms of physiological information.

## Conclusions

The study of oocyte maturation and early development faces a major challenge regarding the physiological relevance of the abundance of total RNA or even poly(A) RNA. The presence of stored and hence inactive maternal RNA that marks developmental stages prior to embryonic genome activation can bias the subsequent interpretation of these measurements. We provide evidence that the study of polyribosomal mRNA offers a better option for studying the physiology underlying gametes and embryonic development especially when the cells are bearing large amounts of stored RNA. The procedure developed in the present study was shown to be robust and efficient for isolating polyribosomal mRNA from small amounts of cells. This polyribosomal mRNA can then be used for downstream transcriptomic studies.

## Methods

All chemicals were from Sigma-Aldrich (St-Louis, MO) unless specified otherwise.

### Oocyte recovery and selection

Germinal vesicle (GV) oocytes were produced as described previously [54] from bovine ovaries collected at a commercial slaughterhouse and transported to the laboratory in a saline solution containing antimycotic agent. Cumulus oocyte complexes (COC) selected on the basis of having at least five layers of cumulus were washed three times in HEPES-buffered Tyrode's medium supplemented with 0.3% bovine serum albumin, 0.2 mM pyruvic acid and 50 μg/ml gentamycin. Groups of approximately 50 COCs were placed in four-well Petri dishes containing 0.5 ml of maturation medium (composed of modified synthetic oviductal fluid medium with 0.8% bovine serum albumin, modified Eagle's medium non essential amino acids, modified Eagle's medium essential amino acids, 1 mM glutamine, 0.5 μg/ml follicle-stimulating hormone, 5 μg/ml luteinizing hormone and 1 μg/ml 17β-estradiol) under 0.5 ml of mineral oil. Germinal vesicle breakdown (GVBD) and metaphase II (MII) oocytes were obtained by incubating COCs at 38.5°C with 5% CO_2 _for 6 and 24 hours respectively. Maturation was stopped upon transfer to PBS-cycloheximide. Cumulus cells were removed by vortexing the tissue in PBS containing 100 μg/ml of cycloheximide to prevent translation and ribosome run off. The denuded oocytes were washed at least three times with PBS-cycloheximide to remove any remaining cumulus cells.

Groups of 75 GV, GVBD or MII oocytes collected for polyribosomal extraction were separated from the PBS-cycloheximide by centrifugation at low speed.

### Carrier preparation

Since a single bovine GV oocyte, even though enriched with stored RNA, may contain as little as 340 pg of total RNA [20] and polyribosomal mRNA represents only a small fraction of this total, conventional extraction methods for obtaining a UV-distribution profile on sucrose density gradient would require an unobtainable number of oocytes. We therefore devised an inert polyribosome support to serve as a marker and carrier in the sucrose gradient, allowing us to observe the distribution of single ribosomes and polyribosomes. RNA extracted from drosophila SL2 cells was used as a carrier because of its phylogenetic distance from cattle.

SL2 cells were cultured at 28°C in complete Schneider's Drosophila Medium (Invitrogen, Carlsbad, CA) containing 10% heat-inactivated foetal calf serum (Hyclone, Logan, UT), 50 U/ml penicillin G and 50 μg/ml streptomycin sulphate (Invitrogen). To enhance translation and therefore increase the number of polyribosomes, these cells were starved for 3 hours in serum-free medium then stimulated for at least 150 min by adding 33% of fresh medium and 6.25% of heat-inactivated foetal calf serum to the medium. Cells were harvested by centrifugation at 1,200 × *g *for 3 min at 4°C in the presence of 100 μg/ml cycloheximide and lysed in polyribosome lysis buffer (50 mM Tris-HCl pH 8.7, 150 mM KCl, 1.25 mM MgCl_2_, 1% IGEPAL, 0.5% deoxycholate, 200 μg/ml cycloheximide, 1000 U/ml Protector™ RNase inhibitor (Roche, Indianapolis, IN) supplemented with complete mini EDTA-free protease inhibitor cocktail tablets (Roche, Indianapolis, IN) followed by triturating. The homogenate was then clarified by centrifugation at 12,000 × *g *for 20 min at 4°C and the absorbance of the supernatant was measured at 260 nm using a Nanodrop ND-1000 spectrophotometer (Nanodrop, Wilmington, DE, USA). For each cross-linking test described below, clarified cytoplasmic extract was prepared from 8 × 10^6 ^cells.

### Chemical cross-linking of *Drosophila *polyribosomes

Formaldehyde was added to clarified cytoplasmic extract to give final concentrations of 0.2% to 3.37%. All cross-linking was done on ice for 45 min. Residual formaldehyde was neutralized by adding 0.1 M glycine [39] or Tris-HCl at concentrations of 0.15 M to 1.5 M with the pH adjusted to 8.7 or 10.9, followed by 20 min on ice. Each cross-linked and neutralized extract was then processed through the steps designed for isolation of polyribosomes from the gradient fractions. Cytoplasmic samples were mixed with guanidium isothiocyanate solution and the RNA fraction was ethanol precipitated overnight. The pellet was further purified by column chromatography (PicoPure, Molecular Devices, Sunnyvale, CA). Cross-linking efficiency was based on the yield of total RNA thus recovered. It was expected that with increased cross-linkage, more RNA would be covalently bound to and eluted with the protein fraction. The RNA-containing fraction eluted from the column was analyzed using a 2100 BioAnalyzer (Agilent, Santa Clara, CA). To determine if the formaldehyde-treated material retained the potential to generate a normal polyribosomal fractionation profile, it was subjected to isokinetic sedimentation in sucrose density gradient as described below and the generated UV profile distribution was compared to one from an untreated sample.

### Oocyte sample preparation

Since the polyribosome lysis buffer is unable to dissolve the bovine zona pellucida, oocytes were strongly vortexed with 1.0 mm zirconia-silica beads (BioSpec, Bartlesville, OK) in 50 μl of passive lysis buffer (Additional File 1). The lysis product was transferred to a clean Eppendorf tube and the beads were discarded. Cell debris were removed by centrifuging at 12,000 × *g *for 20 min. The clarified solution was added to 100 μl of drosophila carrier. Each sample was loaded on a 4 ml linear sucrose gradient.

### Sucrose gradient preparation and centrifugation

The linear sucrose gradient was generated using a SG-15 gradient maker (Hoefer, Holliston, MA, USA) following the manufacturer's instructions with 10% and 60% solutions of sucrose in isotonic buffer made of 150 mM KCl, 1.25 mM MgCl_2 _and 50 mM Tris adjusted to pH 8.7. The linear gradients were kept at 4°C until use. Cytoplasmic extracts were analyzed by sedimentation velocity in the sucrose gradients for 3 h at 34,000 rpm using a SW 60 Ti rotor (Beckman Coulter, Brea, CA, USA). The gradients were processed using a BR-188 Density Gradient Fractionation System (Brandel, Gaithersburg, MD, USA). Fractions of 350 μl were collected with continuous monitoring of absorbance at 254 nm using an Isco UA-6 detector (Teledyne Isco, Lincoln, NE, USA). The RNA-containing fractions were collected directly in 428 μl of 5.25 M guanidinium thiocyanate (pH 5.5) and 3 μl (5 μg/μl) of linear polyacrylamide (Ambion, Austin, Texas, USA) were added to each as an RNA co-precipitant. Isopropanol was then added and the samples were left overnight at -20°C. The polyribosome-containing RNA precipitate thus obtained was then re-suspended in the extraction buffer provided with the RNA extraction PicoPure kit (Molecular Devices, Sunnyvale, CA, USA) and RNA content was assayed using the NanoDrop ND-1000 spectrophotometer. The polyribosomal nature of the isolated fractions was verified by adding 100 mM of EDTA to the lysis buffer and the sucrose gradient solutions to sequester Mg^2+ ^and thereby disrupt any polyribosomes.

### Microarrays

The hybridizations were performed on the custom-made BlueChip v1.3 cDNA microarray. This microarray contains 1153 expressed sequence tags collected from four subtracted libraries made from oocytes and early embryos [55]. For the determination of the optimal microarray hybridization temperature to avoid potential contamination from the carrier (Table 1) two technical replicates were performed for each tested temperature. To test the reproducibility of the polyribosomal isolation, six hybridization samples were prepared representing three biological replicates each performed in dye swap (two technical replicates). To test the method in a biological context (Figures 6 and 7), for each maturation stage, two biological replicates each containing 75 oocytes were used to generate hybridization samples. A total of 12 microarrays were hybridized including a technical replicate for each sample.

RNA samples were amplified through two rounds of *in vitro *transcription using the RiboAmp kit (Molecular Devices, Sunnyvale, CA, USA). Yields of antisense RNA were assayed on the NanoDrop ND-1000 spectrophotometer. For each microarray hybridization sample, 10 μg of antisense RNA was labelled using the ULS aRNA labelling kit (Kreatech, Amsterdam, The Netherlands). To obtain more concentrated and cleaner output, PicoPure columns (Molecular Devices) were used for aRNA purification. The resulting labelled purified probes were heat-denatured at 90°C for 5 min and 50 μl of SlideHyb buffer #1 (Ambion) was immediately added. The slides were hybridized in the SlideBooster hybridization chamber (Advalytix, San Francisco, CA, USA) at the tested temperature for 18 h. Slides were washed twice in low-stringency buffer (2× standard saline citrate (SSC)/0.5% sodium dodecyl sulphate) for 15 min at 60°C. Washes were repeated with high-stringency buffer (0.5× SSC/0.5% sodium dodecyl sulphate) in the same conditions. Slides were then dipped three times in SSC 1× followed by three more dips in H_2_O, spun for 5 min at 1,200 × *g *at room temperature and scanned using a VersArray ChipReader (Virtek, Bio-Rad, Mississauga, ON, Canada) supported by VersArray software (Bio-Rad).

Signal intensity and local background were determined with Array-Pro Analyzer Ver4.5 (Media Cybernetics, Inc., Bethesda, MD, USA). Pearson's correlation coefficients were calculated based on net signal intensities of probes on the microarray that generated positive signals. The same approach was used to determine the optimal microarray hybridization temperature (Table 1). Positive signal threshold values were determined based on a net intensity cut-off value calculated from the background values + 2 standard deviations. For microarrays, data was pre-processed: 1) background was subtracted; 2) intra-array normalization was performed using Loess; 3) inter-array normalization was performed using Quantile.

### RT-PCR

Total RNA was extracted using PicoPure columns (Molecular Devices). An on-column DNase 1 treatment was performed. The resulting RNA samples were reverse transcribed using the qScript Flex cDNA synthesis kit (Quanta, Biosciences, Gaithersburg, MD, USA). The reaction was primed using either an oligo dT (for poly(A) and polyribosomal RNA) or with random primers to target all mRNAs regardless of poly(A) length, in a total reaction volume of 20 μl. For PCR, primer sequences and details are listed in Table 2.

**Table 2:** Description of RT-PCR or PCR primers for examined genes

Gene name	Gene symbol	Primer sequences 5'-3'	Amplicon size (bp)	Annealing/Melting temp. (°C)	Accession number
Cyclin B1	*CCNB1*	F: ACC TGG CAA AGA ATG TGG TC R: GCT GTG CTA GAG TGC TGA TCT TAG	108	60/80	NM_001045872
Cyclin Dependant kinase 1	*CDK1*	F: GAT CCT GCC AAA CGA ATT TCT GGC R: TCT GCT CTT GAC ACA ACA CAG GGA	121	60/78	NM_174016
Oocyte maturation factor MOS	*MOS*	F: CAA AGC ATT GTG CAC TTG GAC CTC R: TGG GTG TAA CAG GCT CTC CTT TGA	190	60/89	XM_590874
Actin beta	*ACTB*	F: CGCCATGGATGATGATATTG R: GGTCATCTTCTCACGGTTGG	363	60/N/A	NM_173979

For standard PCR (Figure 4), the distribution was repeated twice from biologically independent samples. The Nova*Taq *DNA Polymerase (EMD Biosciences, Gibbstown, NJ, USA) was used and the amplification conditions were as follows: Hot start cycle, 10 min at 95°C; 30 PCR cycles (denaturing: 94°C for 30 sec; annealing: 60°C for 30 sec; extension: 72°C for 1 min) followed by a last 10 min extension at 72°C. The PCR products were loaded onto 1.5% agarose gel for migration.

For quantitative PCR, four biologically independent replicates were processed for all treatments and time points. A standard curve was generated using the template from a PCR product purified using the QIAquick PCR Purification Kit (Qiagen, Mississauga, ON, Canada) and quantified with a NanoDrop ND-1000 spectrophotometer. The standard curve consisted of five serial dilutions of the purified PCR products ranging from 0.1 pg to 0.01 fg. Quantification was achieved using the LightCycler FastStart DNA Master SYBR Green I kit (Roche Diagnostics, Laval, QC, Canada) following the manufacturer's recommendations. All reactions were conducted in a LightCycler 1.5 (Roche Diagnostics). Primer annealing and fluorescence acquisition temperatures are listed in Table 2. Specificity of amplification was determined by sequencing the amplicon for each target and by the presence of a single peak on the melting curve. Data normalization could only be accounted by using samples containing the same amount of oocytes. Since quantifications were performed on different RNA populations (i.e. total RNA, poly(A) bearing and polyribosomal mRNA), data normalization could not be performed across these groups. Furthermore, usual housekeeping gene candidates could not be used for data normalization across oocyte maturation stages since their respective transcript abundance been reported to be fluctuating [56,57]. As a consequence, absolute transcript measurements were considered where total variance includes both technical and biological variances.

### Western blot analysis

Oocytes were frozen in groups of 25 in a minimal volume (1-3 μl) of PBS and stored at -80°C. Three pools of each maturation stage were re-suspended in 2× sodium dodecyl sulphate gel loading buffer (100 mM Tris-Cl, 4% w/v sodium dodecyl sulphate, 0,2% w/v bromophenol blue, 20% v/v glycerol, 10% β-mercaptoethanol) and heated to 95°C prior to loading. The samples were separated by SDS-PAGE (12% acrylamide). Proteins were transferred onto a nitrocellulose membrane (NitroBind Cast) using the wet transfer method and transferred proteins stained with Ponceau S red. The membranes were processed for immunoreactions with the primary antibody overnight at 4°C then with secondary antibody under the following conditions: STAT3 (no. 9132, Cell Signaling Technology, Danvers, MA, USA) diluted 1/1,000 - goat anti-rabbit IgG horseradish peroxidase diluted 1/200,000; GSTM3 (no. 74749, Abcam, Cambridge, MA, USA) diluted 1/2,500 - goat anti-mouse IgG horseradish peroxidase diluted 1/100,000; DTX2 (no. 101938, Santa Cruz biotechnology, Santa Cruz, CA) diluted 1/100,000 - anti-rabbit diluted 1/100,000; PTTG1 (no. 3305, Abcam) diluted 1/2,500 - anti-mouse diluted 1/40,000. Each candidate was immunoblotted in parallel with the usual housekeeping genes: β-actin (no. 4967, Cell Signaling Technology) diluted 1/10,000 - anti-rabbit diluted 1/200,000 or α-tubulin (Santa Cruz biotechnology 33999, diluted 1/250) - rabbit anti-goat IgG horseradish peroxidase (diluted 1/200,000)). Determination of the housekeeping gene products was done according to the molecular weight of the protein of interest to avoid overlapping signals. All secondary antibodies came from Invitrogen. Protein expression levels were quantified using GeneTools software (Syngene, Frederick, MD, USA).

### Statistical analysis and microarray data processing

Significant differences were calculated using SAS software (SAS-Institute inc., Cary, NC). One-way ANOVA with Dunnett tests were conducted for all cross-linking tests by using standard extraction as control. Differences were considered statistically significant (*) at the 95% confidence level (P < 0.05) and highly significant (**) at the 99% confidence level (P < 0.01). For Figure 6, RNA abundance data were analyzed with one-way ANOVA using Tukey's multiple comparison test. For Figure 7, protein and polyribosomal RNA levels were analysed with two-way ANOVA since interrelation between both are expected. Data with different letters are significantly different (P < 0.05). When ANOVA criteria were not met (normality and homogeneity of variance), data were transformed to logarithms.

For microarrays, statistical testing was conducted using the statistical significance test from Limma using a Web-based tool, WebArray DB (http://www.webarraydb.org/webarray/index.html). Only candidates with statistically significantly different signal (p < 0.05) and with at least a twofold change were selected.

## Authors' contributions

SS carried out most of the experiments including the development of the carrier's preparation, the method's robustness assessment and all of its testing in biological contexts. She also drafted the manuscript. JPG carried out the preliminary conditions for carrier and oocyte preparation, assessed the linearity of the sucrose gradient, determined the optimal microarray hybridization conditions and initiated the method's reproducibility testing. MHD performed some of the immunoblots. MAS supported the stipend of JPG and directed his academic program. EWK provided the expertise on polyribosomal and help to draft the manuscript. CR conceived the study, participated in its design and coordination, supervised the graduates and helped to draft the manuscript. All authors read and approved the final manuscript.

## Supplementary Material

Additional file 1 **Oocyte disruption using zirconia-silica beads**. The sturdiness of the bovine zona pellucida requires the use of 1 mm zirconia-silica beads to achieve complete cellular disruption. Click here for file

Additional file 2 **Cross-linking the carrier polyribosomes using UV**. A) Time course treatment of UV exposure. The efficiency of the reaction was assessed by measuring the proportion of total RNA recovered following treatments. B-C) Micro-electrophoretic profiles of the total RNA recovered following 5 min (B) or 30 min (C) of UV exposure. **Methods for additional file ****2**. Aliquots (100 μl) of the clarified cytoplasm extract were loaded into compartments of the Lab-Tek II chamber slide system (Nunc, Roskilde, Denmark). The slides were kept at 4°C on a refrigerated aluminum block. Aliquots were exposed to UV (254 nm) in a UVC500 apparatus (Hoefer, Holliston, MA) at a distance of 5 cm using the maximum intensity setting. Samples were removed at different exposure times and mixed with the guanidium isothiocyanate solution used for RNA extraction. Click here for file
