# Supplementary figures and images for: Method to isolate polyribosomal mRNA from scarce samples such as mammalian oocytes and early embryos
Source: BMC Dev Biol. 2011 Feb 15;11:8. doi: 10.1186/1471-213X-11-8 (PMC3055227; doi:10.1186/1471-213X-11-8)

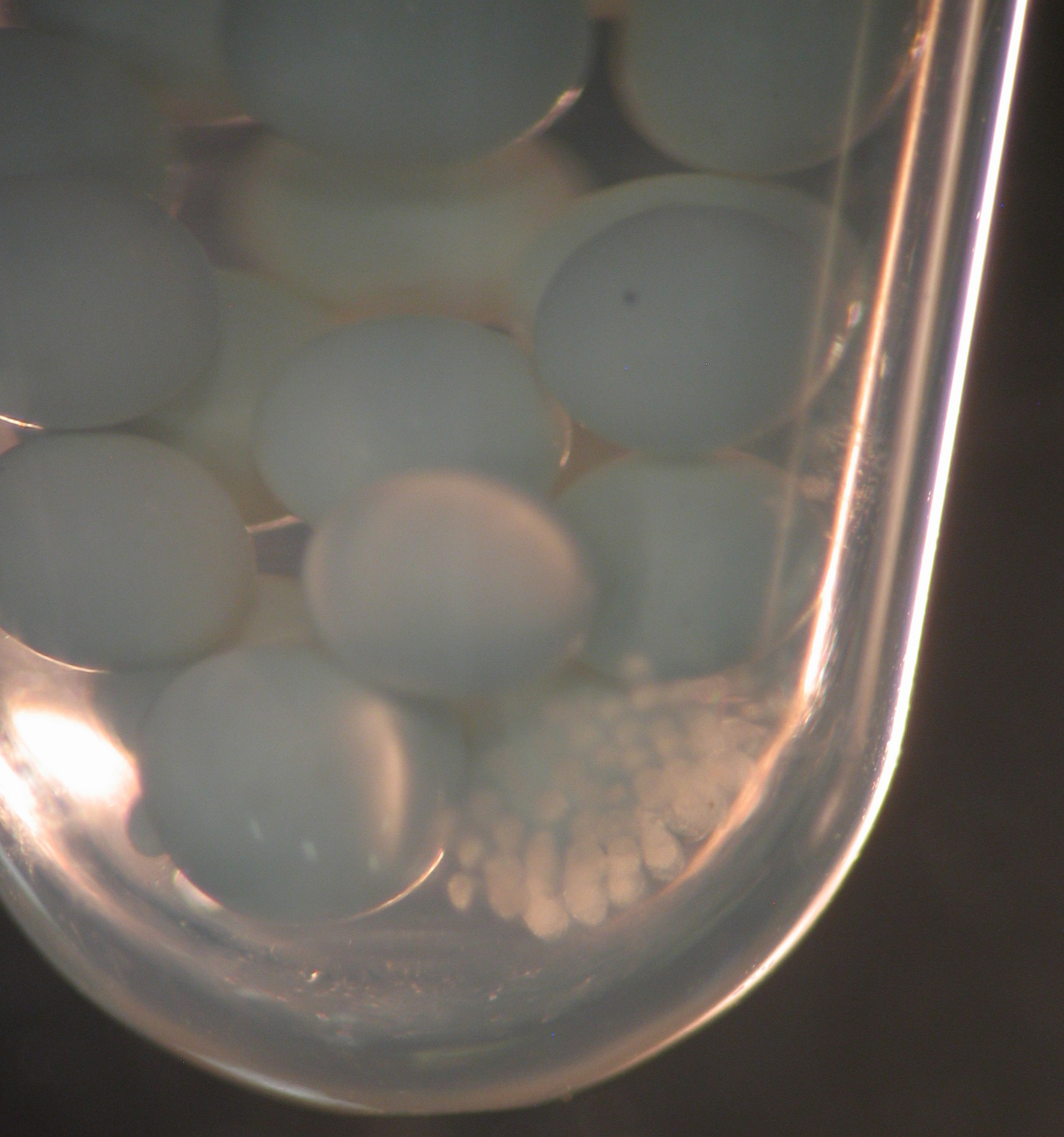

Supplement: Additional file 1 — Oocyte disruption using zirconia-silica beads. The sturdiness of the bovine zona pellucida requires the use of 1 mm zirconia-silica beads to achieve complete cellular disruption. [file 1471-213X-11-8-S1.TIFF]

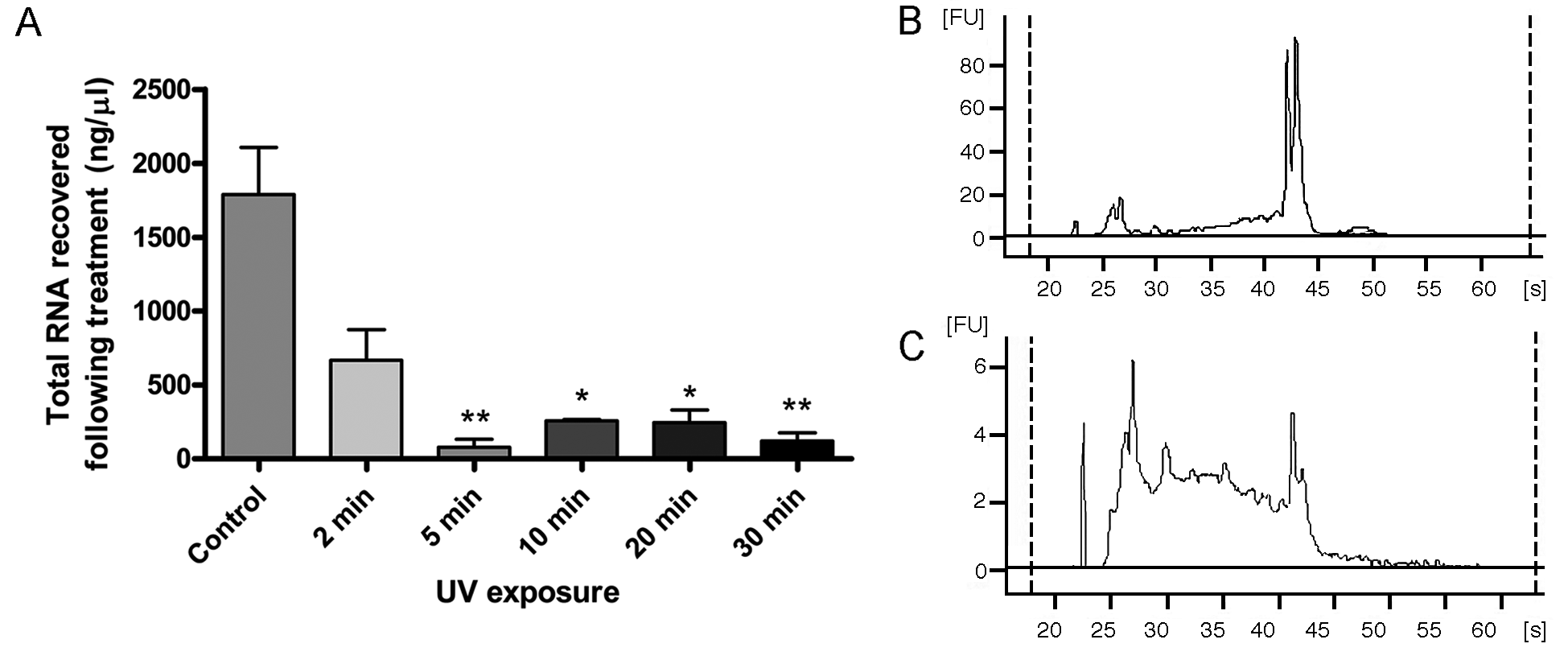

Supplement: Additional file 2 — Cross-linking the carrier polyribosomes using UV. A) Time course treatment of UV exposure. The efficiency of the reaction was assessed by measuring the proportion of total RNA recovered following treatments. B-C) Micro-electrophoretic profiles of the total RNA recovered following 5 min (B) or 30 min (C) of UV exposure. Methods for additional file 2. Aliquots (100 μl) of the clarified cytoplasm extract were loaded into compartments of the Lab-Tek II chamber slide system (Nunc, Roskilde, Denmark). The slides were kept at 4°C on a refrigerated aluminum block. Aliquots were exposed to UV (254 nm) in a UVC500 apparatus (Hoefer, Holliston, MA) at a distance of 5 cm using the maximum intensity setting. Samples were removed at different exposure times and mixed with the guanidium isothiocyanate solution used for RNA extraction. [file 1471-213X-11-8-S2.TIFF]
